# The Moderating Role of Vertical Collectivism in South-Korean Adolescents’ Perceptions of and Responses to Autonomy-Supportive and Controlling Parenting

**DOI:** 10.3389/fpsyg.2018.01080

**Published:** 2018-07-02

**Authors:** Bart Soenens, Seong-Yeon Park, Elien Mabbe, Maarten Vansteenkiste, Beiwen Chen, Stijn Van Petegem, Katrijn Brenning

**Affiliations:** ^1^Department of Developmental, Personality, and Social Psychology, Ghent University, Ghent, Belgium; ^2^Department of Child Development, Ewha Womans University, Seoul, South Korea; ^3^Family and Development Research Centre, Université de Lausanne, Lausanne, Switzerland

**Keywords:** parenting, culture, autonomy, collectivism, adolescence

## Abstract

Research increasingly demonstrates that associations between autonomy-relevant parenting and adolescent adjustment generalize across cultures. Yet, there is still an ongoing debate about the role of culture in these effects of autonomy-relevant parenting. The current study aimed to contribute to a more nuanced perspective on this debate by addressing cultural variability in micro-processes involved in autonomy-relevant parenting and, more specifically, in adolescents’ appraisals of and responses to parental behavior. In this vignette-based experimental study, involving 137 South-Korean adolescents (54% female, mean age = 16 years), we examined whether individual differences in vertical collectivism affect the association between descriptions of potentially autonomy-supportive and controlling parenting practices and (a) appraisals of these practices (in terms of perceived autonomy support and control and experiences of autonomy need satisfaction and frustration), and (b) anticipated responses to these practices (i.e., negotiation, submissive compliance, and oppositional defiance). Participants in the autonomy-supportive condition reported more perceived autonomy support and autonomy satisfaction and lower perceived control and autonomy need frustration than participants in the controlling condition. Collectivism moderated between-vignette effects on perceived control and autonomy need frustration such that the differences between the autonomy-supportive and controlling vignettes were less pronounced (yet still significant) among adolescents scoring higher on collectivism. Collectivism did not moderate effects of the vignettes on the responses to parenting, but yielded a main effect, with collectivism relating to more submissive compliance and less oppositional defiance. Overall, the results suggest that both universal and culture-specific processes are involved in autonomy-relevant socialization.

## Introduction

Several developmental and motivational theories emphasize the key importance of adolescents’ experiences of autonomy – and of parents’ support of autonomy – for adolescents’ psychosocial adjustment (e.g., Zimmer-Gembeck and Collins, z2003; Ryan et al., 2016; Soenens et al., 2018). One prominent theory in this regard is Self-Determination Theory (SDT; Ryan and Deci, 2017), according to which perceived autonomy-supportive parenting is beneficial for adolescent development because it is conducive to their psychological needs for autonomy, relatedness, and competence (Joussemet et al., 2008a; Grolnick and Pomerantz, 2009). SDT-based research has quite systematically documented positive associations between perceived parental autonomy support and adolescents’ well-being, social adjustment, and achievement (Vasquez et al., 2016). Yet, because most of this research was conducted with Western samples, criticisms have been made about the cross-cultural generalization of these associations. In particular, some cross-cultural scholars have raised doubts about whether the effects of parental autonomy support are indeed universal and also play an adaptive role in the development of adolescents raised in a collectivist cultural climate (Rothbaum and Trommsdorff, 2007). Autonomy-supportive parenting may seem to be particularly at odds with vertical collectivism, which entails hierarchical parent–child relationships and parental dominance (Zhai and Gao, 2009).

To contribute to the debate about the cross-cultural relevance of autonomy-supportive parenting, we examined whether adolescents’ vertical collectivist orientation plays a role in how autonomy-supportive (relative to controlling) practices are interpreted and handled. Specifically, among South-Korean adolescents, we examined whether vertical collectivism would affect (a) adolescents’ *appraisals* of potentially autonomy-supportive and controlling practices (in terms of perceived autonomy-support and the interpretation of these practices as facilitating the need for autonomy) and (b) how adolescents respond to these parenting practices (i.e., submissive compliance, oppositional defiance, negotiation). While a number of studies have begun to examine between-country differences in adolescents’ appraisals of autonomy-relevant parenting (Chao and Aque, 2009; Helwig et al., 2014), few studies looked explicitly into the role of individual differences in cultural orientation (see Marbell-Pierre et al., 2017 for an exception). Further, few studies addressed the question of whether cultural orientation affects adolescents’ responses to parental autonomy-relevant behavior (see Chen et al., 2016 for an exception focusing on between-country differences). Overall, this study aims to gain more insight into the micro-processes involved in the role of culture in autonomy-relevant parenting.

### A Self-Determination Theory Perspective on Parental Autonomy Support

According to SDT, people have three inherent and fundamental psychological needs, the satisfaction of which is crucial for their well-being: the needs for autonomy, competence, and relatedness (Deci and Ryan, 2000; Ryan and Deci, 2000, 2017). The need for autonomy takes a central place in the ongoing debate about cross-cultural differences in effects of parenting (Chirkov et al., 2005). Satisfaction of the need for autonomy involves experiences of psychological freedom, volition, and authenticity. When experiencing autonomy, one feels free to act upon the values, goals and interests one wholeheartedly endorses. Frustration of this need, by contrast, manifests in feelings of pressure and obligation. When experiencing autonomy need frustration, one feels forced to act, think, or feel in specific ways (Ryan and Deci, 2000). Numerous studies have shown that the satisfaction of this need is conducive to children’s (e.g., Veronneau et al., 2005) and adolescents’ (Soenens et al., 2007) well-being, whereas its frustration is predictive of ill-being and even increases risk for psychopathology (Bartholomew et al., 2011; Vansteenkiste and Ryan, 2013). While autonomy satisfaction contributes to psychosocial adjustment across different developmental periods, in adolescence in particular, autonomy satisfaction is related to adolescents’ successful negotiation of crucial developmental tasks such as identity development (Luyckx et al., 2009; Soenens and Vansteenkiste, 2011) and emotion regulation (Brenning et al., 2015; Ryan et al., 2016).

In SDT, parents are considered an important contextual source of adolescents’ autonomy-relevant experiences and subsequent psychosocial adjustment (Grolnick et al., 1997). An autonomy-supportive parental style is said to contribute to experiences of autonomy satisfaction, with autonomy-supportive parenting being defined as a parental style that fosters a sense of volition in children (Grolnick et al., 1991; Soenens et al., 2007; Joussemet et al., 2008a). Autonomy-supportive parents try to take the child’s frame of reference, thereby displaying genuine interest in the child’s feelings and thoughts, even when children express negative emotions or display resistance against parental authority. In addition, autonomy-supportive parents encourage initiative, offer meaningful choices whenever possible, and provide a relevant rationale for rules (Koestner et al., 1984; Soenens et al., 2017). Autonomy-supportive parenting can be contrasted with a more controlling parental style, where parents pressure children to think, feel, or behave in particular ways (Grolnick and Pomerantz, 2009; Soenens and Vansteenkiste, 2010).

Controlling parents would, for instance, threaten with punishments or induce feelings of guilt to obtain immediate compliance. In SDT, the term controlling parenting refers to parenting that is pressuring and domineering in nature. Parental psychological control, which is characteristic of parents who pressure children through insidious and manipulative techniques (e.g., guilt-induction, shaming, and love withdrawal; Barber, 1996), is considered a key example of controlling parenting. Controlling parenting, defined as pressuring parenting, is distinct from more constructive parental attempts to regulate adolescents’ behavior (e.g., through clear communication of rules and supervision of behavior). Such parental practices have been referred to as behavioral control (Barber, 1996) or structure (Grolnick and Pomerantz, 2009). Herein, the term controlling parenting is used to refer to pressuring parental practices and not to more constructive parental regulation of behavior.

The notion that autonomy-supportive parenting is beneficial for adolescents’ psychosocial adjustment has received widespread empirical support. Research has shown systematic and robust positive associations between perceived autonomy-supportive parenting and high-quality motivation for behavior in different life domains (e.g., studying and sports), subjective well-being, and academic performance (Soenens and Vansteenkiste, 2005; Joussemet et al., 2008a; Grolnick and Pomerantz, 2009; Vasquez et al., 2016). Increasingly, studies also demonstrate that satisfaction of the need for autonomy accounts for these positive effects of autonomy-supportive parenting (Costa et al., 2016). In contrast, there is mounting evidence that controlling parenting is predictive of ill-being and problem behavior in adolescents (Joussemet et al., 2008b), and that these effects are accounted for by autonomy frustration (Costa et al., 2015; Mabbe et al., 2016).

### Autonomy-Supportive Parenting Considered Through a Cross-Cultural Lens

The robustness of the findings obtained on autonomy-supportive parenting in the West, together with the SDT-based conceptualization of autonomy as a universal need, has raised questions about the generalizability of these findings across cultures. Some scholars voiced skepticism about the presumed universally beneficial role of autonomy-supportive parenting in adolescents’ development and have even claimed that autonomy-suppressive (i.e., controlling) parenting may be functional and adaptive in certain cultural contexts (Chao, 1994; Rothbaum and Trommsdorff, 2007; Grusec, 2008).

A central argument in these cross-cultural criticisms is that autonomy-supportive parenting is at odds with the values emphasized in more collectivist cultures, such as interdependence, harmony, loyalty, obedience, and interpersonal closeness. Autonomy-suppressive (i.e., controlling) parenting would be more functional in collectivist cultural settings because such parenting conveys the importance of loyalty, compliance, and tightly knit family bonds (Rothbaum and Trommsdorff, 2007).

To some extent, these criticisms leveled against the universally beneficial effects of autonomy-supportive parenting are rooted in conceptual confusion about what autonomy (and its support) involves (Vansteenkiste et al., 2005; Ryan and Deci, 2017). In SDT, autonomy is not the same thing as independence (Ryan et al., 2016; Soenens et al., 2018). While independence refers to the degree to which adolescents act without relying on parental input (an orientation contrasted with dependence), autonomy as defined in SDT refers to the intrapersonal experience of volition and self-endorsement of one’s actions (Chen et al., 2013). Even when complying with parental or societal expectations (which involves dependency), adolescents do not necessarily give up their autonomy. Adolescents can experience a sense of volition even when complying with expectations. Critical in this regard is that adolescents come to see the personal relevance of these expectations and accept them as their own (Chirkov et al., 2003). Similarly, parental support for autonomy is different from the promotion of independence (Soenens et al., 2007). In SDT, autonomy-supportive parenting does not merely involve encouraging children to become fully self-reliant. Rather, it involves parental encouragement of children to act upon personally endorsed values and goals.

This differentiated viewpoint on autonomy allows us to reconsider the relation between autonomy and collectivistic values. Parental encouragement of autonomy is indeed at odds with collectivist values when one defines autonomy as independence. To illustrate, Qin et al. (2009) found that United States adolescents showed higher initial levels and stronger increases in independent decision-making in parent–adolescent relationships in the transition from 7th to 8th grade than Chinese adolescents. Moreover, although higher initial levels of independent decision-making related to enhanced emotional functioning in both China and the United States, increases in independent decision-making were only related to increases in well-being among United States adolescents. These findings suggest that adolescents with a collectivist cultural background do not benefit from independence in parent–adolescent relationships to the same extent as adolescents with a more individualistic cultural background. In contrast, autonomy is not at odds with collectivist values when autonomy is defined as volitional functioning. Collectivist practices and values, such as expectations about loyalty, can be communicated in a way that supports children’s volition. For instance, parents can discuss different ways in which these values can be realized, explain the personal importance of these values, and be receptive to the child’s ideas regarding these values. This type of autonomy-supportive style is expected to contribute to adolescents’ self-endorsement of cultural values and to subsequent well-being (Chirkov et al., 2003, 2005).

Research increasingly supports the presumed universal benefits of autonomy-as-volition, with studies in countries all over the world demonstrating that satisfaction of the need for autonomy relates positively to adolescents’ well-being (Chirkov et al., 2005; Vansteenkiste et al., 2006; Yu et al., in press) and that autonomy need frustration predicts ill-being (Sheldon, 2011; Chen et al., 2015b). For instance, Chen et al. (2015b) demonstrated such findings in four countries from different continents (i.e., China, Peru, Belgium, and the United States), each of which was characterized by a markedly different cultural climate. In a follow-up study, these findings were corroborated among poverty stricken Chinese individuals raised in rural areas (Chen et al., 2015a). Similarly, there is mounting evidence that adolescents’ perceptions of autonomy support relate to positive developmental outcomes and that perceptions of controlling parenting are predictive of maladjustment across nations from the Middle-East (Ahmad et al., 2013), Latin America (Chirkov and Ryan, 2001), and East Asia (Wang et al., 2007; Soenens et al., 2012; see Yu et al., 2018 for a review).

### Toward a Nuanced Perspective on the Role of Culture in Parental Autonomy-Support

Do the findings discussed so far imply that culture does not affect autonomy-supportive parenting in any way? No. Culture may still play an important role in adolescents’ appraisals of parental behavior, that is, in their perceptions of parents as being autonomy-supportive or more controlling and in the interpretations of parental behavior as facilitating or hindering autonomy need satisfaction. Furthermore, culture may also play a role in adolescents’ responses to autonomy-relevant parental practices (Soenens et al., 2015; Grusec et al., 2017; Grolnick et al., 2018).

#### Cultural Differences in Appraisals of Parenting

Research has begun to show that adolescents’ cultural background influences their perceptions and interpretations of potentially autonomy-supportive and -suppressive practices (Rudy et al., 2008; Chao and Aque, 2009). In a study with adolescents from Ghana, a West-African country characterized by a predominantly collectivist cultural orientation, Marbell and Grolnick (2013) found that items tapping into parental provision of choice for decision-making were not perceived by adolescents as autonomy-supportive (i.e., as supporting volitional functioning). Rather, the provision of choice was interpreted as the encouragement of independence or even separation, which may possibly conflict with collectivist values such as loyalty and respect. A recent study (Marbell-Pierre et al., 2017) testifies to this culture-specific interpretation of parental choice-provision. This parental practice was only related to positive developmental outcomes among United States adolescents and not among Ghanaian adolescents. Furthermore, it only related to positive developmental outcomes among adolescents that endorsed independent cultural values and not among adolescents that endorsed interdependent values (Marbell-Pierre et al., 2017).

These findings are in line with studies that show that the provision of choice more generally (rather than parental provision of choice specifically) is less motivating among individuals with a collectivist cultural background compared to individuals with a more individualistic cultural background (Iyengar and Lepper, 1999). To explain such findings, Savani et al. (2010) argued and showed that, depending on one’s cultural background, the offer of choice is appraised differently. That is, individuals with an independent self-construal (or disjoint model of agency) tend to construe the offer of choice more easily as autonomy-enabling, while this is less the case for individuals that hold more interdependent self-construals (or conjoint models of agency). Such findings suggest that individuals with a more collectivist background are perhaps less likely to experience an opportunity to select an action as a real choice and, therefore, are less likely to reap the motivational benefits of choice (Patall and Hooper, 2018).

Conversely, there is evidence that items tapping into potentially autonomy-suppressive (i.e., controlling) parental practices are perceived as somewhat more benign in adolescents from collectivist countries, compared to those from individualist countries. When presented with items describing potentially controlling parental behavior, adolescents from collectivist (relative to individualistic) countries reported lower perceived control (Mason et al., 2004) and lower autonomy frustration (Chen et al., 2016). Adolescents from a more collectivist cultural background also evaluated potentially controlling parental behavior less negatively (Helwig et al., 2014), anticipated less feelings of anger (Chao and Aque, 2009), and displayed stronger beliefs that the parent’s control attempts were well-meant (Camras et al., 2012).

In sum, adolescents living in more collectivist cultures appear less inclined to perceive some potentially autonomy-supportive practices (such as choice provision) as actually supporting their volitional functioning. Conversely, these adolescents appear to have a somewhat more benign appraisal of potentially controlling practices. It should be noted, however, that even adolescents from a collectivist cultural background have more benign appraisals of autonomy-supportive parental practices compared to controlling ones (Chen et al., 2016).

#### Cultural Differences in Responses to Parenting

Cultural orientations may not only affect adolescents’ appraisals of parenting but also their responses to autonomy-supportive and controlling parental practices (Soenens et al., 2015). Research with Western adolescents shows that autonomy-supportive and controlling styles of communicating a parental request (e.g., a request to study more) yield different types of responses to the request (Van Petegem et al., 2015a; Van Petegem et al., 2017b). An autonomy-supportive style of communication has been shown to increase the likelihood that adolescents respond to parental requests constructively, for instance by negotiating with parents (i.e., trying to reach a compromise with parents). A more constructive response such as negotiation allows adolescents to stay true to themselves (i.e., to experience autonomy satisfaction) and at the same time creates room for parents to continue to use an autonomy-supportive style of communication in future interactions (Skinner and Edge, 2002). In contrast, a controlling communication style is related to less constructive responses, such as submissive compliance (i.e., passively obeying the request) and oppositional defiance (i.e., doing exactly the opposite of what is expected; Van Petegem et al., 2015a).

Theoretically, it can be reasoned that collectivism plays a role in adolescents’ responses to parental requests. Specifically, adolescents that endorse more collectivist values are likely to refrain from responding in ways that challenge parents’ authority (i.e., negotiation and oppositional defiance) and would be more likely to display submissive compliance because this response is consistent with values of harmony and conformity. A vertical (hierarchical) collectivistic orientation in particular emphasizes parents’ authority and dominant role in child-rearing and means that children’s obedience to their parents should be unconditional (Zhai and Gao, 2009). Thus, it can be expected that adolescents who adopt more vertical collectivistic values would report more submissive compliance and less oppositional defiance.

At the same time, this pattern of responses presumed to be associated with vertical collectivism may become especially evident when adolescents are exposed to a controlling parental style. If a controlling communication style indeed serves to convey the importance of collectivist values (e.g., Rothbaum and Trommsdorff, 2007), adolescents high on collectivism may be more sensitive to the effects of a controlling style and display a pattern of responses consistent with their cultural values. Somewhat consistent with this reasoning, Chen et al. (2016) found that Chinese adolescents were less likely than Belgian adolescents to respond to controlling parental behavior with negotiation and more likely to engage in submissive compliance. Somewhat surprisingly, there were no between-country differences in oppositional defiance. Clearly, more research on this issue is needed, especially at the level of between-person differences in cultural orientation.

### The Present Study

In the current study, we aimed to gain more insight into the role of vertical collectivism in adolescents’ appraisals of and responses to autonomy-supportive and controlling parenting. In doing so, we focused on individual differences in vertical collectivism rather than performing a between-country comparison. There is increasing recognition that within-country differences in cultural orientation are substantial and may even exceed between-country differences (e.g., Oyserman et al., 2002). Even within countries typically categorized as high on collectivism, there is much heterogeneity in individuals’ endorsement of collectivist values and practices. As a result, it is important for researchers to not only look at between-country differences but to also study within-country individual differences in vertical collectivism.

We sampled adolescents from South Korea, a country traditionally described as high on collectivism (Hofstede et al., 2010). Influenced by Confucian philosophy, traditional South-Korean family life is characterized by close family ties and by an emphasis on loyalty toward family values (e.g., Cheah and Park, 2006). Controlling parenting practices (such as shaming) are considered rather appropriate practices to enforce obedience (Han, 1999; Park et al., 2009). However, it is important to note that the impact of traditional Confucian values has waned in recent years (Zhang et al., 2005). Due to effects of globalization, Western values such as independence have also gained prominence in Korean culture. As a consequence, families and individuals within Korea differ widely in the degree to which they adhere to collectivist or more individualistic values. Because of this large within-country heterogeneity in the adoption of cultural values, South-Korea is an interesting country to examine the role of collectivism in parenting.

In this study, we presented South-Korean adolescents with vignettes describing either an autonomy-supportive or controlling style of communicating a parental request (i.e., a request to study more after a poor grade; see Van Petegem et al., 2015a; Chen et al., 2016). An important advantage of this vignette-based experimental design is that it allows for a detailed examination of how a standardized parental statement is interpreted. This design also allows for an examination of how adolescents respond to the parental statements.

Using this methodology, a first goal was to examine the effects of parental communication style and collectivism on South-Korean adolescents’ appraisals of parental behavior (i.e., their perceptions of parenting and needs experiences) and on their responses (i.e., negotiation, submissive compliance, and oppositional defiance). First, we hypothesized that adolescents in the autonomy-supportive condition (compared to those in the controlling condition) would report perceiving more autonomy support and autonomy need satisfaction and less control and autonomy need frustration. Second, we hypothesized that adolescents would report more negotiation, less submissive compliance and less oppositional defiance in the autonomy-supportive (relative to the controlling) condition (Van Petegem et al., 2017b).

A second goal was to examine the interplay between vertical collectivism and experimentally induced communication style on adolescents’ appraisals. We hypothesized that vertical collectivism might dampen differences in appraisals between the autonomy-supportive and controlling styles, with between-condition differences being less pronounced among adolescents scoring high on vertical collectivism.

A third goal was to examine the interplay between vertical collectivism and communication style on adolescents’ responses. We expected the combination of high collectivism and exposure to the controlling style to result in the highest levels of submissive compliance and the lowest levels of negotiation. Regarding oppositional defiance, we expected that the main effect of the controlling (relative to the autonomy-supportive) vignette would be dampened among adolescents that score higher on collectivism. In terms of adolescents’ responses, on the basis of previous research (e.g., Chen et al., 2016), we also expected a main effect of vertical collectivism, with collectivism relating negatively to negotiation and oppositional defiance and positively to submissive compliance.

A fourth goal was to also examine the moderating role of vertical collectivism with regard to adolescents’ appraisals of the parental request. Adolescents’ appraisals are likely to be more strongly related to adolescents’ responses than to the experimentally induced (autonomy-supportive or controlling) situation *per se*. Indeed, it seems likely that adolescents’ responses are determined to a greater extent by their interpretation of the situation than by the situation itself. Thus, it was deemed important to also examine the moderating role of collectivism in the associations between the appraisals and the responses and not only in the associations between the experimentally induced situation and the responses. Again, the hypothesis was (a) that a combination of high levels of collectivism and appraisals of parental behavior as controlling and autonomy need frustrating would predict the highest levels of submissive compliance and the lowest levels of negotiation and (b) that high scores on collectivism would reduce the strength of the associations between appraisals of parental behavior as controlling/autonomy need-frustrating and oppositional defiance.

## Materials and Methods

### Participants and Procedure

Participants were South-Korean high-school students following an academic track. All participants were recruited from the same school (in Seoul) and were in the 2nd year of high school. Although initially 150 adolescents were invited to participate, only 138 adolescents completed the questionnaire and one participant with many missing values was removed from the dataset, resulting in a final sample of 137 adolescents (74 females; 54%). Participants’ mean age was 16.04 years, with very little variance. All participants were either 16 or 17 years old (*SD* = 0.21). Of the participants, 92% came from intact families. Mothers’ and fathers’ mean educational level on a 7-point scale [ranging from 1 = *none*, over 4 = *high school (10–12 years of education)*, to 7 = *graduate school*] was 5.36 and 5.74, respectively, both indicating an average of at least 15 years of education.

The data were collected in 2012. At that time, there was no formal obligation for studies to receive approval from the university’s ethical committee (Ewha Womans University, Seoul). In Korea, regulations about approval from the ethical committee were only in effect from 2014. Because the data collection involved a very straightforward design (correlational data collected with questionnaires) and because the topic of the study was not exceptionally sensitive, no explicit permission was obtained through informed consent forms for parents or adolescents. At the time, it was customary to only ask for permission from the school principal. Next, homeroom teachers were informed about the purpose and method of the study by a researcher. The teachers then distributed the questionnaires to their students. It was made clear to adolescents that participation was voluntary and could be discontinued at any time. Students were also told that their data would be treated confidentially. Students had approximately 45 min to complete the survey.

### Measures

All measures used in this study were translated from English to Korean by the second author, whose mother tongue is Korean and who is fluent in English. While the translation of most items into Korean was straightforward, the first and second author of this paper discussed a few terms with potentially different meanings in the two languages. After agreement upon the meaning of these terms, the final translation was double checked and compared with the original English questionnaire item by item.

#### Vertical Collectivism

Participants first filled out a 5-item scale tapping into vertical collectivism. Items were from two well-known and widely questionnaires, with three items (i.e., “One should do what would please one’s family, even if one detests the activity,” “One should sacrifice an activity that one enjoys very much if one’s family did not approve of it,” and “One should take care of one’s family, even when one has to sacrifice what he/she wants”) taken from Chirkov et al.’s (2003) measure of cultural orientations and with two items (i.e., “Children below 18 should always obey their parents” and “A person should always consider the needs of his family, as a whole, as more important than his own”) taken from the Bardis Familism Scale (Rao and Rao, 1979). As can be seen in **Table 1**, the reliability of this scale was modest, a problem quite common in the assessment of cultural orientations (Singelis, 1994). To further inspect the psychometric properties of this scale, we conducted a principal components analysis (PCA) on the five items. Although this PCA yielded two components with an eigenvalue > 1 (2.03 and 1.20, respectively), the scree plot demonstrated an elbow after the first component, which accounted for 40% of the variance. All items had substantial loadings on this component, ranging between 0.44 and 0.73 (mean loading = 0.63). These results indicate that the scale has a clear one-dimensional and internally coherent structure.

**Table 1:** Descriptive statistics and correlations between the study variables.

	Mean (*SD*)	α	Observed range	1	2	3	4	5	6	7
1. Collectivism	2.75 (0.59)	0.62	1.00–4.80							
2. Perceived autonomy support	3.24 (0.96)	0.91	1.00–5.00	0.05						
3. Perceived control	2.63 (0.86)	0.85	1.00–5.00	–0.08	–0.54***					
4. Autonomy need satisfaction	2.89 (0.85)	0.83	1.00–5.00	0.11	0.63***	–0.57***				
5. Autonomy need frustration	2.99 (0.86)	0.78	1.00–5.00	0.11	–0.46***	0.51***	–0.63***			
6. Negotiation	3.35 (0.87)	0.91	1.00–5.00	0.04	0.17*	–0.19*	0.22**	–0.14		
7. Submissive compliance	2.52 (0.84)	0.85	1.00–4.75	0.48***	–0.08	0.07	–0.05	0.14	–0.15^†^	
8. Oppositional defiance	2.58 (0.83)	0.80	1.00–5.00	–0.29**	–0.14	0.33***	–0.20*	0.26**	–0.09	–0.33***

*^†^*p* < 0.10; ^∗^*p* < 0.05; ^∗∗^*p* < 0.01; ^∗∗∗^*p* < 0.001.*

*All items are measured on a 1–5 Likert scale.*

#### Vignettes

Next, participants read a vignette depicting a hypothetical situation in which the adolescent was interacting with his or her mother. Participants were instructed to imagine that they were in the situation. Specifically, participants first read a description of a situation in which an adolescent comes home from school with poor grades. This situation was followed by a maternal reaction involving a request to study more, which was formulated either in an autonomy-supportive way (e.g., showing empathy, providing a rationale for the request) or in a controlling way. Consistent with a definition of controlling parenting as parenting that is pressuring in nature (Grolnick and Pomerantz, 2009), the language used in the controlling vignette was domineering and involved shaming. The vignettes focused on the academic domain because this life domain is important across cultures and because it represents a domain in which parents are often involved (e.g., Pomerantz et al., 2007). Also, in traditional Asian culture academic success is associated with a greater sense of responsibility and morality (Li, 2005). As such, parental involvement in this area of life is potentially influenced by cultural orientation. Extensive information about the development and the validity of the vignettes, as well as the materials as such, are provided in Van Petegem et al. (2015a). These vignettes were also validated in previous cross-cultural research which compared Belgian and Chinese adolescents (Chen et al., 2016).

Adolescents in two classes were assigned to the autonomy-supportive condition (*N* = 75) and adolescents in two other classes were assigned to the controlling (*N* = 62) condition (thus, we used a between-subjects design) and in each condition we presented one vignette. A number of students in the controlling condition (*n* = 12) did not complete or hand in their questionnaire, resulting in a somewhat smaller number of participants in this condition. This higher incidence of non-completion in the controlling condition is most likely due to the fact that the homeroom teachers in the two classes that were assigned to the controlling condition did not follow-up as well on students’ completion of the questionnaires (relative to the teachers of the two classes assigned to the autonomy-supportive condition). After reading the situation, participants rated their appraisals and anticipated reactions in the situation using scales that were validated in previous vignette-based studies (Van Petegem et al., 2015a; Chen et al., 2016). The literal text of these vignettes can be found in Chen et al. (2016). All items had five response options, ranging from 1 (“*Completely not true*”) to 5 (“*Completely true*”). Reliabilities (Cronbach’s alpha) of all scales are reported in **Table 1**.

#### Perceived Situational Autonomy Support and Control

All items tapping into perceived autonomy-support and control began with the item stem ‘If my mother would react like this, I would feel like ….’ Perceived maternal autonomy support was assessed through four items of the Autonomy Support subscale of the Perceptions of Parents Scale (Grolnick et al., 1991), adapted to the context of the hypothetical situation (i.e., “she allows me to make my own plans for the things I do,” “…she permits me to choose what to do, whenever possible,” “…she is willing to consider my point of view,” and “…she allows me to decide things for myself”). Perceived parental control was assessed through four items from the Psychological Control Scale (Barber, 1996), which were also adapted to the described situation (e.g., “… she is not very sensitive to my needs,” “… she is trying to change how I see things,” “… she is disappointed in me,” and “… she insists upon doing things her way”). The latter four items were also used in Chen et al. (2016).

#### Situational Autonomy Need Satisfaction and Frustration

Participants rated the degree to which they would experience satisfaction or frustration of their basic psychological needs in the situation using an adapted version of the Basic Psychological Need Satisfaction and Frustration Scale (BPNSFS; Chen et al., 2015b). This questionnaire taps into satisfaction and frustration of the needs for autonomy, relatedness, and competence. As our study focused specifically on the issue of adolescent autonomy, we only used the subscales assessing autonomy satisfaction and autonomy frustration (see Van Petegem et al., 2015a for a similar approach). Similarly, these items were preceded by the item stem ‘If my mother would react like this, I would ….’ Four items tapped into adolescents’ experienced autonomy need satisfaction (i.e., “…experience a sense of choice and freedom,” “…feel that I am able to do what I really want,” “…… feel I am able to express who I really am,” and “… feel I can do what really interests me”) and four items tapped into adolescents’ autonomy need frustration (i.e., “…feel forced to do things I wouldn’t choose to do,” “…feel obliged to do certain things,” “…feel pressured to study for this course,” and “… feel like studying for this course is an obligation”).

#### Anticipated Behavioral Responses

Adolescents then reported on how they would respond (behaviorally) to the described situations. We tapped into three responses, that is, oppositional defiance (i.e., a tendency to do exactly the opposite of what parents expect), submissive compliance (i.e., passive adherence to parental expectations), and negotiation (i.e., constructive attempts to reconcile one’s own goals with parental goals). Each of the three responses was assessed with four items. Oppositional defiance was measured with a scale developed and validated by Vansteenkiste et al. (2014). An example item is *“I would rebel against my mother’s request.”* Items for submissive compliance and negotiation were used and validated by Chen et al. (2016). Example items are: *“I would do what she expects from me, even if what she says is not meaningful to me”* (submissive compliance) and *“I would explain to my mother what I think about it”* (negotiation).

## Results

### Descriptive Statistics and Background Variables

Descriptive statistics and correlations between the study variables can be found in **Table 1**. To determine whether some of the background variables were related to the study variables (and had to be controlled for as potentially confounding variables), we examined effects of gender, age, family structure (intact versus non-intact families), and both parents’ educational level on the study variables. To avoid using a variable with very few cases in some of the categories, parents’ educational level was recoded into a variable with only three categories: (a) high school education or lower (representing 20% and 30% of fathers and mothers, respectively), (b) higher education (college or university; representing 62% of both fathers and mothers), and (c) graduate school (representing 18% and 8% of fathers and mothers, respectively). We performed a MANCOVA with gender, family structure, and paternal and maternal educational level as fixed factors, with age as a covariate, and with all study variables as dependent variables. None of the background variables had a multivariate effect on the study variables, Wilk’s Lambda *F*(8,93) = 0.92, *p* > 0.05, $\eta_{p}^{2}$ = 0.08 for gender, Wilk’s Lambda *F*(8,93) = 0.93, *p* > 0.05, $\eta_{p}^{2}$ = 0.07 for family structure, Wilk’s Lambda *F*(8,93) = 0.93, *p* > 0.05, $\eta_{p}^{2}$ = 0.07 for age, Wilk’s Lambda *F*(16,186) = 0.85, *p* > 0.05, $\eta_{p}^{2}$ = 0.08 for paternal educational level, and Wilk’s Lambda *F*(16,186) = 0.83, *p* > 0.05, $\eta_{p}^{2}$ = 0.08 for maternal educational level.

To inspect whether randomization of participants across conditions was successful we examined associations between condition allocation and (a) the background variables and (b) collectivism. We used χ^2^-tests for associations with categorical variables (gender and family structure) and independent samples *t*-tests for associations with continuous variables (age, parental educational level, and collectivism). None of these associations was significant, indicating that the random assignment of participants across conditions was successful.

### Main Effects of Parental Style: Between-Vignette Differences in Appraisals and Responses

We first examined the main effects of condition (autonomy-support versus control) on the appraisals and responses. For this aim, we conducted two MANOVAs with condition: one with the appraisals as dependent variables and one with the responses as dependent variables. While the multivariate effect of condition on the appraisals was significant, Wilk’s Lambda *F*(4,123) = 0.66, *p* < 0.001, $\eta_{p}^{2}$ = 0.34, the multivariate effect on the responses was not, Wilk’s Lambda *F*(3,130) = 0.97, *p* > 0.05, $\eta_{p}^{2}$ = 0.04. Results of the univariate effects of condition on each of the study variables can be seen in **Table 2**. As expected, participants in the autonomy-supportive (relative to the controlling) condition reported higher levels of perceived autonomy support and autonomy need satisfaction and lower levels of perceived control and autonomy need frustration. Unexpectedly, condition did not directly affect any of the responses.

**Table 2:** Means and standard deviations for the experimental conditions.

	Autonomy-supportive	Controlling	*F*	*P*	$\eta_{p}^{2}$
	condition	condition			
Perceived autonomy support	3.52 (0.76)	2.90 (1.05)	15.72	0.00***	0.11
Perceived psychological control	2.28 (0.66)	3.06 (0.89)	33.96	0.00***	0.20
Autonomy need satisfaction	3.27 (0.65)	2.44 (0.85)	41.64	0.00***	0.24
Autonomy need frustration	2.62 (0.68)	3.45 (0.83)	41.50	0.00***	0.24
Negotiation	3.31 (0.88)	3.39 (0.86)	0.27	0.60	0.00
Submissive compliance	2.43 (0.85)	2.62 (0.82)	1.62	0.21	0.01
Oppositional defiance	2.52 (0.81)	2.64 (0.84)	0.72	0.40	0.01

### The Moderating Role of Collectivism in Between-Vignettes Differences in Appraisals

Next, we examined whether collectivism would moderate effects of condition (autonomy-support versus control) on the appraisals. The overall moderating effect of collectivism on between-vignette differences in appraisals was examined with a MANOVA including condition as a fixed factor, collectivism as a covariate, and the condition × collectivism interaction as a predictor. The multivariate effect of the condition × collectivism interaction approached significance in the prediction of the appraisals, Wilk’s Lambda *F*(4,120) = 0.94, *p* = 0.10, $\eta_{p}^{2}$ = 0.06.

Although the multivariate effect was only marginally significant, we still examined interactions between collectivism and condition on the specific appraisals in detail through a series of moderated regression analyses, thereby following the procedures outlined by Aiken and West (1991). Scores for collectivism and condition were centered and an interaction term was computed as the product of both centered scores. Each of the study variables was then regressed on the main effects of condition and collectivism and on their interaction. Results are shown in **Table 3**.

**Table 3:** Interactions between collectivism and condition in the prediction of appraisals and responses to parental behavior (the first coefficients shown are standardized regression coefficients – the second coefficients shown are unstandardized coefficients – with standard errors between brackets).

	Perceived	Perceived	Autonomy	Autonomy	Negotiation	Submissive	Defiance
	Autonomy	Control	Need	Need		compliance	
	Support		Satisfaction	Frustration			
**Step 1**							
Condition	0.32^∗∗∗^	–0.46^∗∗∗^	0.52^∗∗∗^	–0.48^∗∗∗^	–0.03	–0.09	–0.13
(0 = controlling, 1 = autonomy-supportive)	0.61 (0.17)	–0.80 (0.14)	0.87 (0.13)	–0.80 (0.13)	–0.05 (0.16)	–0.16 (0.14)	–0.21 (0.14)
Collectivism	0.08	–0.13	0.12	0.10	0.05	0.47^∗∗∗^	–0.35^∗∗∗^
	0.14 (0.14)	–0.19 (0.12)	0.17 (0.11)	0.14 (0.12)	0.08 (0.14)	0.69 (0.12)	–0.49 (0.12)
*R*^2^	0.10	0.22	0.27	0.24	0.00	0.24	0.13
**Step 2**							
Condition × collectivism	–0.14^†^	0.17^∗^	–0.09	0.16^∗^	–0.08	0.01	0.11
	–0.47 (0.28)	0.49 (0.23)	–0.26 (0.22)	–0.46 (0.23)	0.23 (0.27)	0.02 (0.24)	0.31 (0.24)
Change in *R*^2^	0.02	0.03	0.01	0.02	0.01	0.00	0.01

*^∗^*p* < 0.05; ^∗∗^*p* < 0.01; ^∗∗∗^*p* < 0.001; ^†^*p* < 0.10.*

All main effects of condition were consistent with the findings reported in the preceding paragraph (which included only the main effects of condition). Collectivism did not directly predict any of the appraisals. Finally, we inspected the interactions between condition and collectivism and we found that the interaction term predicted perceived control and autonomy need frustration (but not perceived autonomy-support, autonomy need satisfaction, or any of the three responses). The two significant interactions were inspected by testing simple slopes using the approach proposed by Dawson (2014). Effects of condition were tested at two values of the moderator, that is, at 1 SD below the mean (reflecting low levels of collectivism) and at 1 SD above the mean (reflecting high levels of collectivism). Both interactions indicate that between-condition differences in perceived control and autonomy need frustration are most pronounced in adolescents scoring low on collectivism and least pronounced in adolescents scoring high on collectivism. Specifically, there was a pronounced effect of condition on both perceived control (gradient of simple slope = 1.08, *t*-value = 5.56, *p* < 0.001) and autonomy need frustration (gradient of simple slope = 1.07, *t*-value = 5.65, *p* < 0.001) among adolescents scoring low on collectivism.

**FIGURE 1:**
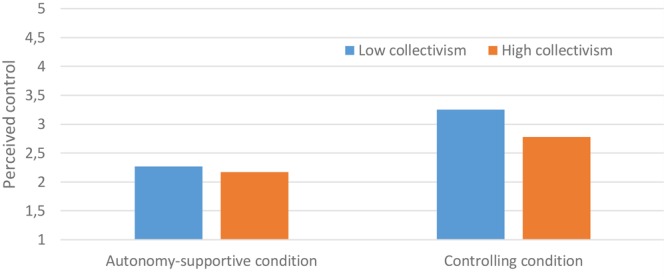
Interaction between collectivism and condition in the prediction of perceived control.

Although these between-condition differences were attenuated among adolescents scoring high on collectivism, they were still significant, with adolescents in the controlling condition reporting more perceived control (gradient of simple slope = 0.511, *t*-value = 2.63, *p* = 0.01) and autonomy need frustration (gradient of simple slope = 0.54, *t*-value = 2.83, *p* < 0.01) than adolescents in the autonomy-supportive condition. The interaction between collectivism and condition in predicting perceived control is displayed graphically in **Figure 1**. The shape of the interaction predicting autonomy need frustration is similar. Consistent with our hypotheses, these findings suggest that adolescents high on collectivism have less negative appraisals of the controlling condition than adolescents low on collectivism. Notably, even adolescents high on collectivism report more positive appraisals (perceived autonomy and autonomy need satisfaction) and less negative appraisals (perceived control and autonomy need frustration) in the autonomy-supportive condition compared to the controlling condition.

### The Moderating Role of Collectivism in Between-Vignettes Differences in Responses

Then, we examined whether collectivism would moderate effects of condition (autonomy-support versus control) on the responses, again using a MANOVA including condition as a fixed factor, collectivism as a covariate, and the condition × collectivism interaction as a predictor. The multivariate effect of the condition × collectivism interaction was not significant in the prediction of the responses, Wilk’s Lambda *F*(3,127) = 0.99, *p* = 0.86, $\eta_{p}^{2}$ = 0.01.

Regression analyses testing effects of condition, collectivism, and their interaction on each of the separate responses (**Table 3**) revealed that collectivism displayed a number of direct associations with the responses. Largely in line with expectations, collectivism was positively predictive of submissive compliance and negatively predictive of oppositional defiance. However, the interaction between condition and collectivism did not predict any of the responses. Thus, collectivism did not moderate any of the associations between condition and adolescents’ responses.

### The Moderating Role of Collectivism in the Relations Between Appraisals and the Responses

A final set of analyses tested the possibility that collectivism moderates associations between adolescents’ appraisals of the situation and their responses. As shown in **Table 1**, adolescents’ appraisals of the experimentally induced situations were related significantly to the responses. These correlations suggest that adolescents’ responses are guided more strongly by their appraisals of the situation than by the experimental condition to which they were exposed. Therefore, it was deemed important to also examine the moderating role of collectivism in the associations between the appraisals and the responses.

For this aim, we ran a final set of regression analyses. Interaction terms were computed between collectivism and each of the four appraisals and these interaction terms were entered (together with the main effects) in the prediction of each of the three responses, resulting in 12 regression analyses. The results of these analyses are shown in **Table 4**.

**Table 4:** Interactions between collectivism and appraisals in the prediction of responses to parental behavior (the first coefficients shown are standardized regression coefficients – the second coefficients shown are unstandardized coefficients – with standard errors between brackets).

	Negotiation	Submissive	Defiance
		compliance	
**Step 1**			
Perceived autonomy support	0.18^∗^	–0.12	–0.11
	0.16 (0.08)	–0.11 (0.07)	–0.09 (0.07)
Collectivism	0.03	0.48^∗∗∗^	–0.30^∗∗^
	0.04 (0.13)	0.69 (0.11)	–0.40 (0.12)
*R*^2^	0.03	0.24	0.10
**Step 2**			
Perceived autonomy support × collectivism	–0.10	0.08	0.09
	–0.12 (0.11)	0.09 (0.10)	0.10 (0.10)
Change in *R*^2^	0.01	0.01	0.01
**Step 1**			
Perceived control	–0.20^∗^	0.12	0.32^∗∗∗^
	–0.20 (0.09)	0.12 (0.08)	0.30 (0.07)
Collectivism	0.04	0.48^∗∗∗^	–0.30^∗∗^
	0.06 (0.13)	0.71 (0.11)	–0.42 (0.11)
*R*^2^	0.04	0.24	0.20
**Step 2**			
Perceived control × collectivism	0.22^∗^	0.02	0.00
	0.32 (0.13)	0.03 (0.11)	0.01 (0.11)
Change in *R*^2^	0.05	0.00	0.00
			
**Step 1**			
Autonomy need satisfaction	0.23^∗∗^	–0.11	–0.17^∗^
	0.24 (0.09)	–0.12 (0.08)	–0.17 (0.08)
Collectivism	0.03	0.49^∗∗∗^	–0.28^∗∗^
	0.04 (0.13)	0.70 (0.11)	–0.39 (0.11)
*R*^2^	0.05	0.24	0.12
**Step 2**			
Autonomy need satisfaction × collectivism	–0.08	0.02	0.10
	–0.11 (0.14)	0.03 (0.12)	0.14 (0.12)
Change in *R*^2^	0.01	0.00	0.01
**Step 1**			
Autonomy need frustration	–0.16^†^	0.10	0.30^∗∗^
	–0.17 (0.09)	0.10 (0.08)	0.29 (0.08)
Collectivism	0.09	0.47^∗∗∗^	–0.36^∗∗∗^
	0.13 (0.13)	0.68 (0.11)	–0.50 (0.11)
*R*^2^	0.03	0.25	0.19
**Step 2**			
Autonomy need frustration × collectivism	0.02	–0.03	–0.01
	0.03 (0.14)	–0.05 (0.12)	–0.01 (0.12)
Change in *R*^2^	0.00	0.00	0.00

*^∗^*p* < 0.05; ^∗∗^*p* < 0.01; ^∗∗∗^*p* < 0.001; ^†^*p* < 0.10.*

We first inspected the main effects of the appraisals on adolescents’ responses. Consistent with our expectations, we found that, while perceived autonomy support and autonomy need satisfaction were related positively to negotiation (although the association with perceived autonomy-support was only marginally significant), perceived control and autonomy need frustration were related to defiance. None of the appraisals were directly related to submissive compliance. Also in line with expectations and confirming the preceding set of regression analyses, collectivism was related positively to submissive compliance and negatively to defiance.

We then inspected interactions between the appraisals and collectivism in the prediction of the responses. Out of the 12 tested interactions, only one reached significance. That is, perceived control and collectivism interacted to predict negotiation. This interaction was difficult to interpret because the association between perceived control and negotiation was non-significant both at low levels of collectivism (*b* = 0.05, *p* > 0.05) and at high levels of collectivism (*b* = -0.31, *p* > 0.05). Although there was a tendency for perceived control to relate to less negotiation among adolescents scoring high on collectivism, this tendency was not significant. Overall, there was little evidence that collectivism moderated associations between adolescents’ appraisals and responses.

## Discussion

The question of whether effects of autonomy-relevant parenting generalize across cultures has elicited debate and even controversy (Rothbaum and Trommsdorff, 2007; Chao and Aque, 2009). Although research has addressed this issue by comparing different countries, considerable differences exist in the cultural orientation of individuals living within the same country. Accordingly, it is also important to look into the role of within-country differences in cultural orientation. This study addressed the role of individual differences in collectivism in both the way adolescents appraise parental behavior and in the way adolescents respond to (perceived) parental practices. We focused on vertical collectivism in particular because this type of collectivism most strongly emphasizes children’s obedience to adult authority. As such, this type of collectivism may seem to be most at odds with an autonomy-supportive parental style.

### Appraisals of Autonomy-Supportive and Controlling Parenting

A first important set of findings indicated that collectivism moderated some of adolescents’ appraisals of the vignettes representing autonomy-supportive and controlling parental behavior. The autonomy-supportive, relative to the controlling, vignette was generally perceived to be more autonomy-supportive and less controlling and came with greater experiences of autonomy need satisfaction and less need frustration. However, adolescents scoring high, relative to those scoring low, on collectivism differentiated less strongly (in terms of two of the four appraisal outcomes) between these two types of parental behavior. Specifically, among adolescents scoring high on collectivism, differences between the two types of vignettes were less pronounced in terms of perceived control and autonomy need frustration. These results are consistent with previous studies which showed that adolescents living in Asian countries generally have a more benign interpretation of potentially autonomy-suppressive parental practices (Chao and Aque, 2009; Camras et al., 2012; Helwig et al., 2014). A possible explanation is that controlling practices convey the importance of collectivist values (such as obedience and family loyalty) and may reflect more parental care (Rothbaum and Trommsdorff, 2007). Specifically, in families that strongly endorse these values, adolescents may perceive the use of controlling practices as somewhat more legitimate and informational, resulting in a more benign appraisal. The current study is among the first to demonstrate this effect with an explicit measure of inter-individual differences in collectivism.

Still, it is important to note that, even among adolescents high on collectivism, the controlling vignette was perceived as more controlling and as more strongly thwarting the need for autonomy compared to the autonomy-supportive vignette. Accordingly, collectivism did not cancel out effects of autonomy-supportive (versus controlling) parenting on adolescents’ appraisals. It only affected the degree to which potentially autonomy-suppressive parenting represented a threat to the need for autonomy. Also, collectivism did not moderate effects of condition on perceived autonomy-support and autonomy satisfaction. Thus, adolescents high on collectivism were equally as likely as adolescents low on collectivism to perceive the autonomy-supportive condition as an opportunity to get their need for autonomy satisfied. Overall, these findings are inconsistent with an extreme relativistic viewpoint on parenting, according to which any type of parental behavior may give rise to any type of interpretation, depending on cultural differences (Soenens et al., 2015). The current findings suggest that there are real and important average associations between the things parents actually do and say (operationalized in this study through the vignettes) and the way these parental behaviors are appraised by adolescents. Although individual differences in collectivism may affect the strength of these associations, an autonomy-supportive parental approach generally resulted in more need-satisfying perceptions and experiences compared to a controlling approach among all adolescents.

Finally, it should be noted that the multivariate effect of the interaction between condition and collectivism did not reach conventional levels of significance in the prediction of the overall set of appraisals. Moreover, the effect size of the few interactions obtained was small. Together, these findings indicate that in this study collectivism only played a modest role in moderating between-vignette differences in adolescents’ appraisals.

### Responses to Autonomy-Supportive and Controlling Parenting

A second set of findings dealt with the role of collectivism in adolescents’ responses to parental behaviors. Collectivism did not moderate between-vignette differences in adolescents’ responses nor associations between adolescents’ appraisals of the vignettes and their responses. The associations between adolescents’ appraisals and their responses in the current sample of South-Korean adolescents were strikingly similar to associations obtained among adolescents from Western samples (Skinner and Edge, 2002; Van Petegem et al., 2015a, 2017a). When adolescents perceived the parental behavior as supportive of the need for autonomy, they were more inclined to negotiate constructively with parents about the request. Instead, when they perceived the parental behavior as controlling and as a threat to their need for autonomy, they were inclined to defy against the parental request and do exactly the opposite.

Overall, these findings are in line with the theoretical prediction that, while perceived autonomy-support creates conditions for adolescents to interact with parents in an authentic, flexible, and constructive fashion, perceived autonomy thwarting elicits defensive reactions in adolescents. While the latter defensive reactions may be undertaken to restore one’s sense of threatened autonomy, their reactive nature is likely to give rise to feelings of inauthenticity (Van Petegem et al., 2015b) and may even result in an increasing cascade of coercive parent–adolescent interactions (Kuczynski, 2003; Vansteenkiste et al., 2014).

The finding that the associations between adolescents’ appraisals and defiance does not depend on individual differences in collectivism was somewhat unexpected, yet largely in line with findings obtained by Chen et al. (2016). These authors examined between-country differences (between China and Belgium) in associations between appraisals and various responses, including oppositional defiance. Most strikingly, perceived control and need frustration were positively predictive of oppositional defiance irrespective of individual differences in collectivism. Although these findings point to a potential universal process, they do not preclude the possibility that the manifestation of adolescents’ defiant responses differs depending on their cultural orientation. Indeed, there may be (culturally determined) variability in the way adolescents defy the parental requests, an issue that could be addressed in future qualitative research. For instance, adolescents high on collectivism may more often feel an inclination to defy parental authority without putting this inclination into practice or these adolescents may defy parental authority in less visible ways (e.g., by pretending to study instead of explicitly refusing to study).

Further, in the present study, individual differences in collectivism did not alter the relation between perceived control/need frustration and either submissive compliance or negotiation, a finding that contrasts with those reported by Chen et al. (2016), who examined between-country differences. One reason for this could be that the concept of submissive compliance in particular requires further differentiation. In future research, it will be important to distinguish between submissive compliance (which reflects a pressured type of submission to parental standards) and accommodation, which reflects a more willing and self-endorsed type of acceptance of parental standards (Skinner and Edge, 2002). With accommodation, adolescents understand the personal relevance of the parental requests and are more likely to concede to these requests with a sense of volition (rather than pressure). Because accommodation – possibly in combination with mild negotiation of the parental request to arrive at a consensus – provides more opportunities for adolescents to stay true to themselves (while still conforming to family-based standards), it might be more beneficial than submissive compliance to adolescents’ well-being, even among individuals growing up in a collectivist context (e.g., Chirkov et al., 2005; Chen et al., 2013). Perhaps collectivism does play a more prominent moderating role in associations between appraisals of parental behavior and compliance when differentiating more clearly between dysfunctional and constructive types of compliance.

Another reason for the somewhat inconsistent findings about the moderating role of collectivism in adolescents’ responses could be that other factors (beyond collectivism) play a stronger role in associations between appraisals and responses. One potential other factor could be individuals’ history of parenting. For instance, adolescents with a history of high levels of autonomy-supportive parenting might tend to use more negotiation when faced with occasional unfair and need thwarting parental behaviors (Van Petegem et al., 2017b). Clearly, there is a need for further research about the moderating role of culture and other relevant factors in associations between appraisals of parental behaviors and adolescents’ responses.

### The Contribution of Vertical Collectivism

While there was little evidence for a moderating role of collectivism in associations between (actual or perceived) communication style and responses, collectivism did display two interpretable and strong main effects on adolescents’ responses. That is, adolescents scoring higher on collectivism were more inclined in general (i.e., across autonomy-supportive and controlling conditions) to comply passively with parental requests and less inclined to defy the parental requests. These findings are consistent with the notion that collectivism, and vertical collectivism in particular, entails strict obedience to parental authority (Zhai and Gao, 2009).

The strong association between collectivism and submissive compliance is particularly noteworthy. It raises the question of whether adolescents high on vertical collectivism interact with their parents in a suboptimal fashion. By complying with parental requests even when disagreeing with the requests, these adolescents risk giving up on their personal goals, thereby experiencing inauthenticity (Skinner and Edge, 2002). Research in Western samples has indeed shown that passive compliance with parental requests forecasts developmental problems (Crittenden and DiLalla, 1988; Kuczynski and Kochanska, 1990). An important question to be addressed in future research is whether the meaning and consequences of submissive compliance differs in collectivist (relative to individualistic) cultural contexts, thereby distinguishing (as noted earlier) clearly between submissive compliance and accommodation. Qualitative research may be ideal to examine the subtleties and nuances in adolescents’ reasoning about submissive compliance and accommodation. Possibly, there is a thinner line between both strategies among adolescents that endorse more collectivist values. Because the preferences, interests, and goals of adolescents high on collectivism are intertwined strongly with those of their family members, parental standards may be followed for a combination of both more pressured reasons (i.e., submissive compliance) or more self-endorsed reasons (i.e., accommodation).

### Limitations

Although this study contributed to previous research in a number of ways (e.g., by relying on a vignette-based approach and by including an individual differences measure of collectivism), it had a number of limitations. First, the reliability of the measure for collectivism was modest. Because at the time of data collection we were unaware of a highly reliable scale tapping specifically into vertical collectivism, we compiled items from a number of different existing questionnaires. Future research would do well to use more elaborate and internally consistent measures of vertical collectivism. Moreover, this measure tapped into only one specific type of collectivism. Future research would do well to include a multi-dimensional assessment of various features of collectivism (Vignoles et al., 2016).

Second, assignment to the two conditions occurred at the level of classes (with two classes being assigned to each of the two conditions). As a consequence of this procedure, there was an imbalance between the two conditions in terms of the completion of the questionnaires, with 12 adolescents in the controlling conditions not completing the questionnaire. Although a test for randomization revealed no differences between participants in the two conditions in terms of socio-demographic characteristics and collectivism, future research would do well to randomize within classes, thereby also avoiding the possibility that class-level dynamics affect the results.

Third, the items were translated thoughtfully by a highly experienced researcher fluent in English and with Korean as her mother tongue. The reliabilities were quite similar to those reported in previous research with Belgian and Chinese participants (Van Petegem et al., 2015a; Chen et al., 2016). Still, because we did not adopt a formal translation-back translation procedure and because no direct comparison was made between the current Korean sample and samples of adolescents with a different cultural background, we cannot ascertain that Korean adolescents attributed the same meaning to the items as adolescents from other countries or cultures. Chen et al. (2016) demonstrated the factorial invariance of the scales used in the current study between Belgian and Chinese adolescents. Future research would do well to formally test the factorial invariance of these scales with Korean participants as well.

Fourth, the sample used in this study was quite homogeneous in terms of family structure and educational level. Adolescents came from families who lived in an urban area and had a rather high level of education. The homogeneity of the sample may have reduced the variance of the scores on the measure for vertical collectivism and may, as such, be partly responsible for the limited number of effects obtained with collectivism in this study. More generally, it is unclear whether the findings would generalize to more heterogeneous samples and to samples of economically more disadvantaged families in particular. Also, in rural areas people may endorse collectivist values to stronger and more extreme degrees. Thus, a stronger test of our hypotheses could be conducted by sampling adolescents from rural areas.

Fifth, by examining adolescents’ responses to hypothetical vignettes we cannot ascertain that adolescents would actually respond this way in real-life situations. This is particularly the case with oppositional defiance, a response that may be inhibited by situational constraints (including the threat of severe consequences for disobedient behavior). Further, social desirability may have affected adolescents’ responses, with adolescents high on collectivism in particular possibly being more inclined to provide overly positive ratings of parental behavior. Future observational research would do well to also examine adolescents’ responses in their actual interactions with their parents (either within the home context or in a laboratory). This research could then examine associations between actual (i.e., observed and coded) parental behaviors and adolescents’ responses.

Sixth, we only examined adolescents’ appraisals and responses in the academic domain. This domain was chosen because parents stay involved in this domain during adolescence (Pomerantz et al., 2007) and because it has a moral connotation in collectivist contexts (Li, 2005). However, adolescents’ appraisals and responses might differ in other social domains, with cultural orientation playing an important role herein. Thus, future research could examine the role of cultural differences (including collectivism) in adolescents’ responses to parental rule-setting in more sensitive and personal life domains such as friendships and social media use. Adolescents scoring high on collectivism might be more inclined to concede to parental standards in these more personal domains than adolescents scoring high on individualism. Social domain theory is an interesting framework in this regard because this theory deals with the boundaries of parental authority and with adolescents’ reasoning about legitimate parental authority (Smetana, 2006).

Seventh, it is important for future research to separate different aspects of autonomy-supportive parenting and to examine cultural differences in specific components of parental autonomy-support. Marbell-Pierre et al. (2017) recently showed evidence for cultural differences in effects of parental provision of choice but not in effects of parental empathy and perspective-taking. Possibly, parental perspective-taking has a more proximal relation with adolescents’ need satisfaction (such that it is universally beneficial) than parental provision of choice, which has a more distal relation to psychological need satisfaction. Because the latter practice can be interpreted quite differently depending on individuals’ cultural orientation, its effects may be more culture-specific. Thus, future research would benefit from a differentiated approach to the operationalization of autonomy-relevant parental practices.

Finally, rather than focusing either on between-country differences (as was done typically in previous research) or on within-country (individual) differences in cultural orientation (as was done in the current study), future research would do well to examine both types of differences simultaneously. Indeed, a combined investigation of between-country and within-country cultural differences would yield the most complete picture of the role of culture in effects of parenting. An important advantage of including participants from different cultures and nations would be that the variance in individual-level cultural orientation would increase, thereby providing better opportunities to examine the moderating role of cultural orientation along a broader spectrum of scores. Also, this type of research would allow one to address the question (a) whether between-country differences or individual differences in cultural orientation are more important in determining appraisals and responses to parenting and/or (b) whether between-country differences in adolescents’ appraisals and responses are mediated by individual-level differences in cultural orientation (see Marbell-Pierre et al., 2017, for an example).

## Conclusion

Collectivism was found to affect South-Koreans adolescents’ perceptions of potentially autonomy-supportive (relative to controlling) parental practices to some extent. Adolescents high on collectivism differentiated less strongly between both types of practices in terms of perceived controllingness and autonomy need frustration compared to adolescents low on collectivism. Yet, even the adolescents high on collectivism appraised the autonomy-supportive parental behavior more positively (e.g., reporting less autonomy need frustration and more autonomy need satisfaction) than the controlling behavior. Moreover, adolescents’ appraisals of the parental behavior related to their responses in theoretically meaningful ways, with experiences of autonomy satisfaction predicting a constructive approach (i.e., negotiation) and with experiences of autonomy frustration predicting a more defensive approach (i.e., oppositional defiance). The latter associations were not moderated by individual differences in collectivism. Overall, the findings are in line with a moderate universalistic view on parenting and, in particular, with the notion of ‘universalism without uniformity’ (Schweder and Sullivan, 1993; Wang et al., 2007; Soenens et al., 2015). According to this view, there is room for individual and culturally determined differences in parenting processes that are universally relevant and important.

## Author Contributions

BS and S-YP conceived of the study. S-YP collected the data and BS prepared a first draft of the manuscript. BS and EM performed the statistical analyses. S-YP, EM, BC, MV, SVP, and KB provided feedback and helped writing the manuscript.

## Conflict of Interest Statement

The authors declare that the research was conducted in the absence of any commercial or financial relationships that could be construed as a potential conflict of interest.
